# On the Secale Cornutum, Clavus, or Ergot of Rye

**Published:** 1825-07

**Authors:** Henry Davies

**Affiliations:** Conduit-Street


THE LONDON

### Medical and Physical Journal.

N° 1 OF VOL. LIV.] JULY, 1825. [N° 317.

For many fortunate discoveries in medicine, and for the detection of numerous errors, the world is indebted to the rapid circulation of Monthly Journals; and there never existed any work, to which the aculty, in Europe and America, were under deeper obligations, than to the Medical and Physical Journal of London, now forming a long, but an invaluable, series.—RUSH.

### ORIGINAL COMMUNICATIONS,

### SELECT OBSERVATIONS, &c.

*To the Editors of the London Medical and Physical Journal.*

Gentlemen,—Having been some time in America during the late war, frequent mention was made to me, by the American practitioners, of the effects of ergot, or spurred rye, in producing uterine action. My practice at that time being confined to the wives of the soldiers, among whom protracted labour seldom occurs, I had no opportunity of proving its efficacy; but, since my return to England, being induced to make use of it, I procured some from Dr. Bibby, of New York; who, at the same time, politely furnished me with proper directions for using it; and, having distributed it among several medical practitioners in various parts of the country, as well as my own immediate friends, who have also made known to me the result of their experience, in compliance with their repeated solicitations, and your request, I have collected notes of such cases as are interesting, preceded by such remarks on its natural history, and general reputed effects, as I have been able to collect from every available source. Hoping it may be the means, through your valuable publication, of diffusing a more general knowledge of its properties, I remain yours, faithfully,

HENRY DAVIES.

*Conduit-street; June 1st,* 1825.

### Art. I.—*On the Secale Cornutum, Clavus, or Ergot of Rye.*

It appears that many of the grasses and graniveous plants are subject to a disease, to which vegetable pathologists have given the name of *clavus.* In this disease, one or more grains are usually enlarged or elongated, and project from the spike or panicle to which they belong: they are of an irregular, curved form, something like, but much smaller, than the spur of a cock; of a light and brittle texture, a dark colour, and unpleasant taste; and, as far as experiments have been tried, incapable of germination. The substance thus produced is called, in England and America, the horned or spurred rye; in France, denominated ergot; by medical men, secale cornutum.

Rye, if not the most subject to this disease, has at least more frequently attracted the notice of observers: in particular seasons, it is found in some ears in almost every field of rye; but it is more plentiful in damp grounds, and in seasons when intense heat succeeds to long-continued rain. It is also said to be found more in the borders of fields than in the central parts, and more in grounds newly broken up than in those long cultivated.

There are a variety of conjectures as to its origin: by some it is attributed to the larvæ of insects, which are productive of numerous and variety of diseases in vegetables; of which, galls and oak-apples are familiar examples. Dr. Chapman thinks it may probably arise, like the several sorts of smut or blight, from a mushroom of the genus scleroticum, which is of a parasitic nature: he says, ergot is an elongated excrescence, which fills the place of the seed within the glume, or husk of the rye.

Various diseases are supposed to have originated by feeding on bread made of this diseased rye, and some of a very extensive and alarming description have been spoken of. Of these, the most common is a species of dry gangrene, pervading at the same time entire districts. The account of these diseases is very unsatisfactory, and it would rather appear that a deficiency of the nutritive property, contained in the bread made of the ergoted rye, disposed the persons who fed on it to disease, than that the ergot was the specific cause: neither does it appear to be prejudicial, except when used in large quantities. Ergoted rye is, however, generally considered unwholesome; and rye-bran, possibly from a mixture of this substance, is supposed by farmers to produce miscarriage in cattle.

It is stated to have been used in France, and some parts of America, half a century ago. Of late years it has been called into notice as an article of the materia medica, under the head of emmenagogues, or of abortiva, and appears first to have been introduced to the attention of the medical profession by Dr. Stearns, of New York. It possesses great and peculiar powers of exciting the action of the gravid uterus; and the testimonies in its favour are so many, and so respectable, that they claim for it, at least, a careful trial and a diligent attention to its effects. When exhibited in large doses, it produces generally one long-continued action of the womb, which has been known to last for an hour or

* See Eberle's Materia Medica.

more, until its contents are expelled. When given prematurely, or under improper circumstances, it has occasionally proved injurious to the mother, and more frequently so to the child; but in moderate doses, at short intervals, it increases the vigour of the pains, without producing such excessive and constant action as may become dangerous. In 1774, a Madame Dupille says, she gave it, very finely powdered, in doses of a thimbleful in a table-spoonful of wine, water, or weak broth, and looked for its effects in a quarter of an hour: she only gave it when the presentation was natural, and the labour lingering. In some parts of America, about the same period, the midwives were in the habit of giving *blasted-rye tea*, (infusion of ergot,) for the purpose of quickening the pains of labour, to make them bear down, and thereby accelerate the birth of the child. In some cases it appears to have done good, in others to have been prejudicial.

In those cases where the presentation was natural, and pains inefficient, it quickly caused the patient to bear down, and the child was generally born in an hour after the infusion had been given. It was prejudicial where the presentation was preternatural and the parts rigid; exciting, under these circumstances, delirium and hysteria; the patients continuing in that situation for an hour or two, perhaps till the medicine had ceased to affect the system. The midwives observing this, were induced to lay it aside, especially as the women were unwilling to employ those who used it. Hence it fell into disuse, and appears not to have been employed for a number of years; and a valuable medicine was laid aside and forgotten, for want of discrimination of the proper cases in which it might, or might not, be employed; and, as I said before, we are indebted to Dr. Stearns for introducing it into regular practice. I have used the ergot in several cases, as have some of my medical friends; from which I am enabled to speak positively of its powers in cases of protracted labour, as the subjoined cases will testify: but I am not able to give such satisfactory evidence of its effects in some uterine diseases, as the assertion of some writers would lead us to expect; and there are also some peculiarities, which have occurred in my own practice, that appear at variance with its general character.

Great discrimination is required in the selection of cases to which it may be suited,—as well as caution in its use. A chemical analysis of ergot has been made in France: it is stated to afford carbonic acid and hydrogen, a fœtid principle, a fixed oil in abundance, and a coal difficult to incinerate. Its extract putrifies, and its colouring principle is fixed only by alkalies. In regard to its preparation and exhibition, it may be used either as a powder or infusion: the latter is said to extract all its most active properties; the powder is more speedily administered, but more apt to occasion nausea. The infusion is of a violet colour, nauseous taste, and slightly acrimonious. When given in large doses, it is said to occasion great heat and pain in the hypogastric region, and sometimes to excite vomiting; though I cannot say that I have witnessed the latter effect, except where the stomach has been predisposed to that action.

I have usually given it in doses of a scruple, finely powdered; or the infusion of one drachm to three ounces of boiling water. Its usual effect has been, that, in the space of from three to fifteen minutes after its exhibition, the uterus has been excited to strong and violent action, accompanied by sympathetic effects in the neighbouring parts, (see Roberts' case,) which pains have continued, with little or no intermission or abatement, for a longer or shorter space of time, and then entirely ceased, (see Smith's case.)

It is pretty evident, however, that its effects are entirely confined to the uterus and contiguous parts; as in no one case does it appear that the circulation was affected, however severe the pain might be.

CASE I. *Protracted labour, detailed in* Dr. MERRIMAN's *valuable work on Difficult Parturition.**—Mrs. Howard, aged forty-seven, her seventh labour, was delivered of one child, still-born. Saturday evening, had no return of pain; nor was there any attempt on the part of the uterus to expel the second child, though friction to the abdomen had been used, and an enema administered. She had slept well, and taken her breakfast sitting up in bed. At fifteen hours and a quarter from the birth of the first child, an infusion of one drachm of secale cornutum was given in a small tea-cupful of boiling water. The pains came on in ten minutes after; the child protruded naturally; and, within an hour, the double placenta was delivered, and the labour over. The child was a fine healthy girl, who, with the mother, did remarkably well.

CASE II. *Communicated by* Dr. MERRIMAN.—It was the patient's third labour. The membranes broken, and the waters were discharged during the first twelve hours. At the termination of thirty-six hours, the pains had ceased; the child's head was situated at the brim of the pelvis, with the os uteri soft and dilatable, larger than a crown-piece. An infusion of one drachm of secale in three ounces of water was given: the pains succeeded within five minutes, increasing in force, so that the labour was terminated in less than an hour after giving the secale. The child was a very large boy, apparently still born, but it required no great efforts to restore him to animation.

The Doctor adds, that he made no doubt of a speedy delivery, when once the head had passed through the superior aperture, as the pelvis was very large; but he did not think it probable, when he gave the infusion, that the labour would be over in so short a time.

CASE III.—March 2, 1821, I was requested to see a Mrs. Roberts, of Wellesley-street, Euston-square, who had been in labour upwards of

* This case was communicated [illegible] by Dr. Merriman.—AUTHOR.

thirty-six hours. She had been bled in the early part of the labour; an enema had been also administered; and at five o'clock in the morning, being much fatigued and restless, an opiate had been given. At nine o'clock A.M. when I saw her, she was quite free from pain, but unable to lie down: the os uteri was fully dilated; the head firmly wedged in the brim of the pelvis, lying transversely, presenting a puffy tumor with a smooth surface. She said she had not made water for eighteen hours. Though I could feel no urine in the bladder through the abdominal parietes, I was anxious that the bladder should be evacuated, but was prevented by the head from passing the catheter. The parts were puffy, but not hot; her pulse was eighty-eight. At half-past ten o'clock, I gave her an infusion of secale, (one drachm to three ounces of water:) in about five minutes after, she had a sharp pain; said she wanted to make water, and that she must go to the night-chair. Immediately after, she cried out with an uncommonly severe pain in the back; at eleven o'clock the child was expelled, not very animated. There was a cessation of pain for half an hour, when the placenta was expelled and withdrawn from the vagina; and the whole process was over by twelve o'clock A.M.

3d, 4th, 5th, and 6th March.—The mother quite well. The child had some difficulty of respiration, apparently as if from mucus in the fauces; but he took the breast well, and was a fine infant.

On the 7th March, the mother had an attack of pneumonia, for which she was bled.

End of March.—Mother and child both in excellent health.

CASE IV.—Thursday, August 30. — Smith, ætat. twenty-eight; first child; had been ill since the Sunday evening previous, but labour only commenced on Tuesday morning. She was much depressed in her spirits, from having seen her sister die in labour in the same house. She appeared to have had every remedial means used.

Seven o'clock A.M.—Her pulse was firm, but she was much fatigued and impatient, and bathed in sweat; her abdomen extremely sore to the touch. Head of the child lying high at the brim of the pelvis, (which was somewhat confined,) and situated rather diagonally; membranes whole; os uteri thick, not completely effaced; the belly was extremely tense; pains frequent, feeble, and of no avail. The membranes were ruptured, in order to diminish the tumor; she took some refreshment; her bowels and bladder were attended to; but, at the end of two hours, no alteration was apparent in the advancement of the head.

Quarter before nine o'clock.—Gave an infusion of secale (one drachm to three ounces of water): it had no effect for twenty minutes after; there was then a slight pain, with a disposition to go to stool, but the pain was not any thing equal to what I had before observed from the exhibition of the secale.

Twenty-five minutes after nine o'clock, (forty minutes after the first dose,) I gave her a second dose of infusion, made from the same portion of the secale. Pains continued. The head made some progress; brought the occiput within range of the pubis; first one ear, then the second, was perceptible. The progress was extremely slow; the woman much exhausted, and impatient.

Between ten and eleven o'clock, I applied the forceps, and delivered the child, which was still born, and had been apparently dead for some time. The placenta was retained, from inactivity of the uterus; delivered by the hand, one o'clock.

Evening.—The patient had had a quiet sleep. She had a favourable recovery.

CASE V.—Mrs. Roberts was again in labour, September 28, 1822; attended by Mr. Hallion; and was delivered, after a long, protracted, and severe labour, by the aid of the secale, which operated in ten minutes from the period of taking it. Mother and child both quite well.

Mr. Hallion says, in consequence of Mrs. Roberts changing her residence, he was prevented attending her in her next labour; which she describes as the most severe of all, having continued for the space of two days and three nights; she was attended by three medical gentlemen, and delivered by instruments; the child was less than any of the former, and in a bad state of health.

CASE VI.—Mrs. Gravel was two days and a night in severe labour; was much exhausted; the child's head was impacted; delivery accomplished in twelve minutes after the exhibition of the secale. Child and mother did well.

CASE VII.—Mrs. R—, after a labour of eighteen hours, and the uterine efforts were quite exhausted, was delivered of a fine child, by aid of the secale. Child and mother both well.

CASE VIII.—Mrs. L— had been in severe labour for twenty hours. During the last three hours, the severity of the pains began to cease, and she was much exhausted. At twenty minutes before eight o'clock, gave her an infusion of one scruple of secale in two ounces of water. The pulse began to rise, and the pains to re-act. The os uteri had been perfectly dilated for five hours previous, with the head of the child stationary.

Ten minutes before eight, gave a second dose, of the same proportions, which increased the pains to a great degree; but still the child was not advanced.

Twenty-five minutes after eight, gave another scruple. Pains still increased: the head began to descend, but the tension and rigidity of the perineum and external parts were so great, that delivery could not be effected.

At twenty minutes before nine, gave a fourth scruple, which produced violent uterine contraction, and the head was expelled five minutes after: it was much elongated, and considerably depressed in some parts. The child was very large, and still born; but the mother had not felt the motion of it for eight hours previous to its delivery. The mother did well, and was up on the seventh day.

CASE IX.—A. T., aged forty-two years; her first labour. Membranes broke on Wednesday morning, August 14th, at four o'clock. Was visited in the course of the day: bowels open; pains slight; passed a restless night; continued all day (Thursday) in the same state.

Friday morning, pains regular, but short. On the evening of Friday, os uteri a little dilated.

Saturday, 17th, at nine o'clock A.M.—The head passed the brim, but pains not sufficient to expel the child. Gave an infusion of the secale, (one drachm to three ounces of water,) at three o'clock P.M.; and, in half an hour, the pains increased considerably, and bore down with much force till the child was born, at ten o'clock P.M., six hours after the infusion was given. Child still-born.

CASE X.—Wednesday, August 7th, 1822. Mrs. P., a little healthy woman, aged forty-three; first child. Waters were discharged between three and four o'clock; but pains did not come on till twelve hours after, continuing, with intervals of fifteen minutes, till noon next day. The os uteri not fully dilated; vertex presenting; soft parts disposed to give way.

At two o'clock, head had passed through the os uteri, the soft parts relaxing kindly; the head descended slowly in the pelvis; but the pains became weaker, with longer intervals.

At six o'clock, scarcely any pain; no alteration in the situation of the head. At twenty minutes after six, gave an infusion of one drachm of secale in three ounces of water: in about eight minutes a pain came on, followed by others, increasing in strength and frequency, until forty minutes after seven o'clock, when the child was born. The pelvis was rather small; the child full sized; the cranial bones firm and unyielding, not lapping over; the head much elongated. Child still-born.

The placenta was attached to the fundus uteri, which had contracted upon it, and the whole of the uterus between the os and placenta was in a state of firm and spastic constriction, which I was not enabled to overcome until three hours after the birth of the child, notwithstanding the administration of opiates. The mother did well, and has since had a good natural labour.

CASE XI.—Anne Wall, ætat. twenty-nine, residing at No. 31, South Molton-street; first child. Was in labour thirty-six hours; the os uteri rather rigid. Bled her ad ℥x. at two o'clock. At seven, the parts more fully dilated: the head presented naturally. Waited till ten P.M., then gave her one scruple of secale in powder, which had an immediate effect: she had scarcely taken it five minutes, when violent bearing-down pains came on. The process of labour went on naturally, and she was delivered at twelve o'clock on Sunday night, May 22d, of a still-born male infant. The placenta was retained for a short time.

[To be continued.]

---

ART. II.—*Pathology of Inflammatory Diseases, &c. founded on the Blood's Vitality.* By JOHN JONES, Esq.

