# Erratum in the Present Number

**Published:** 1825-07

**Authors:** 


ERRATUM *in the present Number.*

Page 4, for "communicated *by* Dr. Merriman *to* me," read "*by* me *to* Dr. M."

